# Supplementary material for: Metabolic Profiling of Pregnant Women with Obesity: An Exploratory Study in Women at Greater Risk of Gestational Diabetes
Source: Metabolites. 2022 Sep 29;12(10):922. doi: 10.3390/metabo12100922 (PMC9612230; doi:10.3390/metabo12100922)
Supplement: Supplementary file 1 [file metabolites-12-00922-s001.zip › metabolites-1874990-supplementary.pdf]

Text S1. Methodology for the nuclear magnetic resonance (NMR) Spectroscopy platform used for metabolic profiling

The NMR methodology is based on measurement of three molecular windows from each serum sample using different pulse programs (Soininen *et al.*, 2009, 2015). Two of the spectra (LIPO and LMWM; low-molecular-weight-metabolites) were acquired from the native serum (conducted at 37° C) and one spectrum from serum lipid extracts (LIPID window, at 22° C). NMR spectra are measured using Bruker Avance III spectrometer which operates at 500 or 600 MHz. The LIPO window represents a standard spectrum of human serum demonstrating broad overlapping resonances coming from lipid molecules in lipoprotein particles while the LMWM window includes signals from several low-molecular-weight molecules. This spectrum is recorded using a relaxation-filtered pulse sequence which dampens most of the broad macromolecule and lipid signals to allow detection of small solutes.

-Table S1: Description of spectral capture

Window	Number of transients	Technique
**LIPO**	8	NOESY-presat pulse sequence (mixing time of 10ms and water peak suppression)
**LMWM**	24	Carr-Purcell-Meiboom-Gill (78ms T_2-filter and fixed echo delay of 403μs)
**LIPID**	32	Standard 1D spectrum

-QC and output

Absolute metabolite quantification was automated from the NMR spectra. To resolve the difficulties related to highly overlapping spectral data, a ridge regression model was used for the quantification of each metabolite. Quantification models for the lipoprotein lipid data were calibrated using high performance liquid chromatography methods, and cross-validated against NMR-independent lipid data. Both low molecular weight metabolites and lipid extract measures were quantified as mmol/l based on regression modelling calibrated against a set of manually fitted metabolite measures. Iterative line-shape fitting analysis (PERCH NMR software, PERCH Solutions Ltd., Kuopio, Finland) was used to quantify the calibration data. As a result of experimental variation in the lipid extraction protocol, absolute quantification cannot be directly determined for the lipid extract measures. Thus, total cholesterol quantified from the native serum LIPO spectrum was used to scale the serum extract metabolites.

-References

Soininen, P. *et al.* (2009) 'High-throughput serum NMR metabonomics for cost-effective holistic studies on systemic metabolism', *The Analyst*. Analyst, 134(9), pp. 1781–1785. doi: 10.1039/B910205A.

Soininen, P. *et al.* (2015) 'Quantitative Serum Nuclear Magnetic Resonance Metabolomics in Cardiovascular Epidemiology and Genetics', *Circulation: Cardiovascular Genetics*. Lippincott Williams & WilkinsHagerstown, MD, 8(1), pp. 192–206. doi: 10.1161/CIRCGENETICS.114.000216.

Table S2: Conventional analytical methods

Analyte	Sample	Method	Platform	CV (%)[a]
Insulin	Plasma	Electrochemiluminescence immunoassay	Roche, Cobas e411	<10.3
C-peptide	Serum	Electrochemiluminescence immunoassay	Roche, Cobas e411	<6.2
HbA1c	Whole blood	Turbidimetric inhibition immunoassay	Roche, Cobas c311	<1.4
Fructosamine	Plasma	Colorimetric, nitroblue tetrazolium	Roche, Cobas c311	<3.4
gGT	Plasma	Enzymatic, colorimetric	Roche, Cobas c311	<3.9
ALT	Plasma	Enzymatic, spectrophotometric	Roche, Cobas c311	<3.1
AST	Plasma	Enzymatic, spectrophotometric	Roche, Cobas c311	<2.1
SHBG	Serum	Electrochemiluminescence immunoassay	Roche, Cobas e411	<7.9
hs-CRP	Plasma	Particle enhanced immunoturbidimetric	Roche, Cobas c311	<7.1
Leptin	Plasma	Enzyme-linked immunosorbent assay	R and D Systems	<2.0
Adiponectin	Plasma	Enzyme-linked immunosorbent assay	R and D Systems	<6.9
Ferritin	Plasma	Particle enhanced immunoturbidimetric	Roche, Cobas c311	<1.7
IL-6	Plasma	Enzyme-linked immunosorbent assay	R and D Systems	<9.8
tPA antigen	Plasma	Enzyme-linked immunosorbent assay	Asserchrom tpa (Stago)	<5.7
Vitamin D	Serum	Electrochemiluminescence immunoassay	Roche, Cobas e411	<11.2
hPL	Serum	Enzyme-linked immunosorbent assay	R and D Systems	<5.0

[a] highest coefficient of variation from either time point 1 or 2 quoted. CV= coefficient of variation; gGT= γ-glutamyl transferase; ALT= alanine aminotransferase; AST= aspartate aminotransferase; SHBG= sex hormone binding globulin; hs-CRP= high sensitivity C-reactive protein; IL-6= interleukin-6; tPA-antigen= tissue plasminogen activator antigen; hPL= human placental lactogen.

Table S3: SD difference in NMR metabolites between high-risk women with and without GDM measured at timepoint 1

Metabolites	β [a]	95% CI		*p*-value **(FDR)**
**Total lipids**				
Total lipids in chylomicrons and extremely large VLDL [b]	0.189	-0.043	0.421	18.093
Total lipids in very large VLDL [b]	0.172	-0.061	0.405	12.062
Total lipids in large VLDL [b]	0.171	-0.060	0.403	7.933
Total lipids in medium VLDL	0.161	-0.103	0.426	9.373
Total lipids in small VLDL	0.049	-0.214	0.313	23.211
Total lipids in very small VLDL	- 0.221	-0.521	0.079	4.021
Total lipids in IDL	-0.287	-0.588	0.014	1.444
Total lipids in large LDL	-0.281	-0.581	0.018	1.324
Total lipids in medium LDL	-0.272	-0.572	0.027	1.340
Total lipids in small LDL	-0.272	-0.571	0.027	1.223
Total lipids in very large HDL	-0.281	-0.539	-0.023	0.489
Total lipids in large HDL	-0.123	-0.384	0.138	4.822
Total lipids in medium HDL	0.269	-0.031	0.569	0.991
Total lipids in small HDL	0.387	0.096	0.678	0.105
**Particle diameter**				
Mean diameter for VLDL particles	0.251	0.002	0.499	0.522
Mean diameter for LDL particles	0.030	-0.256	0.316	8.517
Mean diameter for HDL particles	-0.287	-0.533	-0.041	0.221
**Apolipoproteins**				
Apolipoprotein A-1	-0.065	-0.344	0.214	5.859
Apolipoprotein B	-0.148	-0.434	0.138	2.659
Ratio of apolipoprotein B to apolipoprotein A-1	-0.099	-0.372	0.174	3.879
**Cholesterol**				
Serum total cholesterol	-0.244	-0.542	0.055	0.846
Total cholesterol in VLDL	-0.026	-0.303	0.250	6.298
Total cholesterol in chylomicrons and extremely large VLDL[b]	0.145	-0.097	0.387	0.847
Total cholesterol in very large VLDL[b]	0.137	-0.105	0.379	0.916
Total cholesterol in large VLDL[b]	0.147	-0.088	0.382	0.747
Total cholesterol in medium VLDL	0.083	-0.186	0.352	1.806
Total cholesterol in small VLDL	-0.074	-0.355	0.206	1.966
Total cholesterol in very small VLDL	-0.265	-0.566	0.036	0.272
Remnant cholesterol (non-HDL, non-LDL -cholesterol)	-0.159	-0.450	0.130	1.977
Total cholesterol in IDL	-0.288	-0.587	0.010	0.394
Total cholesterol in LDL	-0.289	-0.589	0.010	0.378
Total cholesterol in large LDL	-0.288	-0.586	0.011	0.178
Total cholesterol in medium LDL	-0.285	-0.585	0.015	0.194
Total cholesterol in small LDL	-0.298	-0.599	0.003	0.166
Total cholesterol in HDL	-0.085	-0.356	0.185	3.354
Total cholesterol in HDL2	-0.081	-0.349	0.187	3.332
Total cholesterol in HDL3	-0.102	-0.396	0.192	2.876
Total cholesterol in very large HDL	-0.279	-0.538	-0.021	0.101
Total cholesterol in large HDL	-0.149	-0.404	0.106	0.728
Total cholesterol in medium HDL	0.241	-0.064	0.546	0.346
Total cholesterol in small HDL	0.089	-0.224	0.403	1.610
**Cholesteryl esters**				
Esterified cholesterol	-0.301	-0.597	-0.004	0.264
Cholesterol esters in chylomicrons and extremely large VLDL[b]	0.092	-0.159	0.343	1.301
Cholesterol esters in very large VLDL[b]	0.126	-0.122	0.373	0.864
Cholesteryl esters in large VLDL[b]	0.128	-0.110	0.366	0.780
Cholesteryl esters in medium VLDL	0.009	-0.265	0.285	2.479
Cholesteryl esters in small VLDL	-0.129	-0.416	0.159	0.965
Cholesteryl esters in very small VLDL	-0.262	-0.559	0.035	0.217

Cholesteryl esters in IDL	-0.266	-0.562	0.029	0.193
Cholesteryl esters in large LDL	-0.275	-0.573	0.022	0.170
Cholesteryl esters in medium LDL	-0.285	-0.585	0.016	0.153
Cholesteryl esters in small LDL	-0.304	-0.606	-0.002	0.115
Cholesteryl esters in very large HDL	-0.276	-0.534	-0.018	0.085
Cholesteryl esters in large HDL	-0.140	-0.396	0.115	0.652
Cholesteryl esters in medium HDL	0.245	-0.061	0.550	0.263
Cholesteryl esters in small HDL	-0.017	-0.329	0.294	2.094
**Non-esterified cholesterol**				
**Free cholesterol**	-0.279	-0.579	0.021	0.369
Free cholesterol in chylomicrons and extremely large VLDL[b]	0.195	-0.040	0.429	0.232
Free cholesterol in very large VLDL[b]	0.146	-0.090	0.383	0.493
Free cholesterol in large VLDL[b]	0.161	-0.071	0.394	0.376
Free cholesterol in medium VLDL	0.155	-0.113	0.422	0.549
Free cholesterol in small VLDL	0.018	-0.253	0.289	1.899
Free cholesterol in very small VLDL	-0.261	-0.569	0.048	0.203
Free cholesterol in IDL	-0.332	-0.635	-0.029	0.066
Free cholesterol in large LDL	-0.321	-0.623	-0.019	0.075
Free cholesterol in medium LDL	-0.279	-0.579	0.021	0.137
Free cholesterol in small LDL	-0.256	-0.556	0.044	0.189
Free cholesterol in very large HDL	-0.287	-0.546	-0.027	0.061
Free cholesterol in large HDL	-0.176	-0.428	0.076	0.332
Free cholesterol in medium HDL	0.217	-0.085	0.520	0.303
Free cholesterol in small HDL	0.426	0.131	0.721	**0.009***
**Triglycerides**				
Serum total triglycerides	0.124	-0.146	0.394	1.930
Triglycerides in VLDL	0.177	-0.088	0.443	0.968
Triglycerides in chylomicrons and extremely large VLDL[b]	0.201	-0.032	0.434	0.169
Triglycerides in very large VLDL[b]	0.183	-0.048	0.415	0.222
Triglycerides in large VLDL[b]	0.183	-0.047	0.414	0.214
Triglycerides in medium VLDL	0.197	-0.068	0.462	0.266
Triglycerides in small VLDL	0.129	-0.132	0.391	0.591
Triglycerides in very small VLDL	-0.028	-0.309	0.253	1.492
Triglycerides in IDL	-0.164	-0.472	0.144	1.452
Triglycerides in LDL	-0.173	-0.481	0.136	1.299
Triglycerides in large LDL	-0.185	-0.497	0.127	0.426
Triglycerides in medium LDL	-0.193	-0.502	0.116	0.381
Triglycerides in small LDL	-0.091	-0.383	0.201	0.925
Triglycerides in HDL	0.092	-0.202	0.387	2.501
Triglycerides in very large HDL	-0.090	-0.397	0.216	0.953
Triglycerides in large HDL	-0.073	-0.367	0.219	1.044
Triglycerides in medium HDL	0.266	0.021	0.510	0.055
Triglycerides in small HDL	0.192	-0.069	0.454	0.247
**Phospholipids**				
Total phospholipids	-0.066	-0.353	0.220	2.934
Phospholipids in VLDL	0.054	-0.209	0.318	3.022
Phospholipids in chylomicrons and extremely large VLDL[b]	0.186	-0.047	0.419	0.189
Phospholipids in very large VLDL[b]	0.159	-0.071	0.391	0.281
Phospholipids in large VLDL[b]	0.160	-0.071	0.392	0.278
Phospholipids in medium VLDL	0.151	-0.112	0.414	0.411
Phospholipids in small VLDL	0.055	-0.203	0.314	1.056
Phospholipids in very small VLDL	-0.266	-0.569	0.037	0.132
Phospholipids in IDL	-0.304	-0.604	-0.003	0.206
Phospholipids in LDL	-0.238	-0.532	0.055	0.464
Phospholipids in large LDL	-0.267	-0.564	0.029	0.116
Phospholipids in medium LDL	-0.213	-0.503	0.077	0.227
Phospholipids in small LDL	-0.187	-0.476	0.102	0.314
Phospholipids in HDL	0.039	-0.241	0.318	3.125
Phospholipids in very large HDL	-0.285	-0.541	-0.028	**0.045***

Phospholipids in large HDL	-0.093	-0.359	0.174	0.732
Phospholipids in medium HDL	0.268	-0.029	0.567	0.113
Phospholipids in small HDL	0.447	0.160	0.733	**0.003***
Total phosphoglycerides	-0.128	-0.408	0.152	1.504
Ratio of triglycerides to phosphoglycerides	0.155	-0.104	0.413	0.931
Phosphatidylcholine and other cholines	-0.156	-0.431	0.118	0.993
Sphingomyelins	-0.258	-0.540	0.024	0.270
Total cholines	-0.189	-0.465	0.085	0.634
**Fatty acids**				
Total fatty acids	-0.063	-0.345	0.218	0.948
Estimated degree of unsaturation	-0.202	-0.457	0.053	0.172
22:6, docosahexaenoic acid	-0.021	-0.302	0.257	1.242
18:2, linoleic acid	-0.253	-0.542	0.035	0.119
Omega-3 fatty acids	-0.054	-0.338	0.229	0.984
Omega-6 fatty acids	-0.204	-0.495	0.087	0.232
Polyunsaturated fatty acids	-0.185	-0.476	0.105	0.288
Monounsaturated fatty acids; 16:1, 18:1	0.020	-0.251	0.292	1.201
Saturated fatty acids	-0.018	-0.296	0.259	1.208
**Fatty acid ratios**				
22:6 docosahexaenoic acid to total fatty acids	0.009	-0.188	0.207	1.219
18:2 linoleic acid to total fatty acids	-0.285	-0.521	-0.048	**0.024***
Omega-3 fatty acids to total fatty acids	-0.034	-0.282	0.215	1.044
Omega-6 fatty acids to total fatty acids	-0.238	-0.485	0.009	0.077
Polyunsaturated fatty acids to total fatty acids	-0.220	-0.461	0.021	0.096
Monounsaturated fatty acids to total fatty acids	0.124	-0.045	0.292	0.193
Saturated fatty acids to total fatty acids	0.181	-0.071	0.433	0.202
**Glycolysis related**				
Glucose	0.502	0.198	0.806	**0.001***
Lactate	0.051	-0.180	0.284	0.830
Pyruvate	0.225	-0.059	0.509	0.151
Citrate	-0.003	-0.297	0.291	1.216
**Amino acids**				
Alanine	-0.099	-0.346	0.147	0.522
Glutamine	-0.323	-0.582	-0.064	**0.018***
Glycine	-0.185	-0.443	0.073	0.192
Branched chain amino acids				
Isoleucine	0.106	-0.177	0.389	0.561
Leucine	0.114	-0.157	0.385	0.487
Valine	0.122	-0.156	0.400	0.459
Aromatic amino acids				
Phenylalanine	0.226	-0.048	0.499	0.124
Tyrosine	-0.017	-0.303	0.268	1.053
Histidine	-0.172	-0.409	0.066	0.180
**Ketone bodies**				
Acetoacetate[b]	0.283	-0.014	0.579	0.071
3-hydroxybutyrate[b]	-0.005	-0.294	0.284	1.109
**Inflammatory marker (NMR)**				
Glycoprotein acetlys, mainly a1-acid glycoprotein	0.260	-0.007	0.528	0.063
**Other (NMR)**				
Creatinine	-0.246	-0.503	0.011	0.068
Albumin	0.150	-0.124	0.424	0.316
Acetate[b]	0.003	-0.253	0.259	1.090
**Conventionally measured analytes**				
**Glucose homeostasis**				
HbA1c	0.218	-0.029	0.466	0.091
Insulin [b]	0.200	-0.047	0.448	0.122
C-peptide[b]	0.273	0.026	0.520	**0.033***
Fructosamine	0.131	-0.140	0.402	0.364
**Liver markers**				
gGT[b]	0.252	0.005	0.499	**0.048***

ALT[b]	-0.050	-0.297	0.196	0.722
AST[b]	-0.025	-0.276	0.225	0.922
SHBG	-0.313	-0.564	-0.061	**0.015***
**Adipokines**				
Leptin[b]	-0.013	-0.237	0.211	1.001
Adiponectin[b]	- 0.406	-0.658	-0.154	**0.002***
**Inflammatory markers**				
hs-CRP[b]	-0.027	-0.269	0.215	0.857
IL-6[b]	-0.309	-0.576	-0.041	**0.025***
tPA-antigen[b]	0.206	-0.076	0.489	0.156
Ferritin[b]	0.209	-0.049	0.469	0.115
**Miscellaneous**				
Vitamin D [b]	-0.078	-0.346	0.191	0.576
hPL[c]	-0.131	-0.388	0.126	0.319

* False Discovery Rate (FDR) corrected *p* value <0.05 shown in bold [a] beta coefficient [b] analyte log transformed [c] analyte z score. GDM= gestational diabetes mellitus; gGT= γ-glutamyl transferase; ALT= alanine aminotransferase; AST= aspartate aminotransferase; SHBG= sex hormone binding globulin; hs-CRP= high sensitivity C-reactive protein; IL-6= interleukin-6; tPA-antigen= tissue plasminogen activator antigen; hPL= human placental lactogen; HDL= high-density lipoprotein; IDL= intermediate-density lipoproteins; LDL= low-density lipoprotein; VLDL= very low-density lipoproteins.
Data are adjusted for maternal BMI, parity, ethnicity, age, education and neonatal sex.

Table S4: SD difference in NMR metabolites between high-risk women with and without GDM measured at timepoint 2

Metabolites	β [a]	95% CI		*p*-value **(FDR)**
**Total lipids**				
Total lipids in chylomicrons and extremely large VLDL [b]	0.277	0.021	0.532	5.508
Total lipids in very large VLDL [b]	0.222	-0.023	0.468	6.156
Total lipids in large VLDL [b]	0.193	-0.051	0.436	6.480
Total lipids in medium VLDL	0.153	-0.125	0.433	11.300
Total lipids in small VLDL	-0.066	-0.319	0.186	19.602
Total lipids in very small VLDL	-0.355	-0.615	-0.094	0.216
Total lipids in IDL	-0.486	-0.753	-0.218	**<0.001***
Total lipids in large LDL	-0.498	-0.765	-0.230	**<0.001***
Total lipids in medium LDL	-0.505	-0.772	-0.238	**<0.001***
Total lipids in small LDL	-0.514	-0.780	-0.248	**<0.001***
Total lipids in very large HDL	-0.374	-0.643	-0.105	0.103
Total lipids in large HDL	-0.278	-0.550	-0.006	0.608
Total lipids in medium HDL	0.126	-0.163	0.417	4.860
Total lipids in small HDL	0.192	-0.083	0.466	1.979
**Particle diameter**				
Mean diameter for VLDL particles	0.352	0.089	0.615	0.097
Mean diameter for LDL particles	0.266	-0.006	0.539	0.567
Mean diameter for HDL particles	-0.346	-0.603	-0.089	0.086
**Apolipoproteins**				
Apolipoprotein A-1	-0.349	-0.617	-0.080	0.089
Apolipoprotein B	-0.317	-0.572	-0.061	0.128
Ratio of apolipoprotein B to apolipoprotein A-1	-0.151	-0.420	0.118	2.430
**Cholesterol**				
Serum total cholesterol	-0.515	-0.778	-0.251	**<0.001***
Total cholesterol in VLDL	-0.139	-0.392	0.113	2.040
Total cholesterol in chylomicrons and extremely large VLDL[b]	0.183	-0.075	0.441	0.574
Total cholesterol in very large VLDL[b]	0.178	-0.071	0.427	0.555
Total cholesterol in large VLDL[b]	0.159	-0.082	0.401	0.658
Total cholesterol in medium VLDL	0.014	-0.252	0.280	3.035
Total cholesterol in small VLDL	-0.264	-0.510	-0.018	0.113
Total cholesterol in very small VLDL	-0.404	-0.671	-0.136	**0.010***
Remnant cholesterol (non-HDL, non-LDL -cholesterol)	-0.327	-0.581	-0.073	0.085
Total cholesterol in IDL	-0.501	-0.769	-0.233	**<0.001***
Total cholesterol in LDL	-0.525	-0.796	-0.254	**<0.001***
Total cholesterol in large LDL	-0.513	-0.783	-0.244	**<0.001***
Total cholesterol in medium LDL	-0.529	-0.801	-0.258	**<0.001***
Total cholesterol in small LDL	-0.546	-0.819	-0.273	**<0.001***
Total cholesterol in HDL	-0.291	-0.570	-0.012	0.255
Total cholesterol in HDL2	-0.268	-0.547	0.010	0.354
Total cholesterol in HDL3	-0.397	-0.675	-0.118	**0.029***
Total cholesterol in very large HDL	-0.349	-0.618	-0.079	**0.032***
Total cholesterol in large HDL	-0.269	-0.537	-0.001	0.142
Total cholesterol in medium HDL	0.105	-0.189	0.400	1.370
Total cholesterol in small HDL	-0.394	-0.692	-0.095	**0.028***
**Cholesteryl esters**				
Esterified cholesterol	-0.514	-0.777	-0.250	**<0.001***
Cholesterol esters in chylomicrons and extremely large VLDL[b]	0.091	-0.169	0.352	1.345
Cholesterol esters in very large VLDL[b]	0.179	-0.074	0.433	0.443
Cholesteryl esters in large VLDL[b]	0.137	-0.105	0.379	0.704
Cholesteryl esters in medium VLDL	-0.101	-0.359	0.157	1.150
Cholesteryl esters in small VLDL	-0.341	-0.592	-0.090	**0.021***
Cholesteryl esters in very small VLDL	-0.387	-0.656	-0.118	**0.013***

Cholesteryl esters in IDL	-0.471	-0.736	-0.206	**0.002***
Cholesteryl esters in large LDL	-0.499	-0.767	-0.232	**<0.001***
Cholesteryl esters in medium LDL	-0.531	-0.803	-0.259	**<0.001***
Cholesteryl esters in small LDL	-0.548	-0.822	-0.273	**<0.001***
Cholesteryl esters in very large HDL	-0.349	-0.618	-0.081	**0.026***
Cholesteryl esters in large HDL	-0.257	-0.525	0.011	0.139
Cholesteryl esters in medium HDL	0.124	-0.171	0.419	0.933
Cholesteryl esters in small HDL	-0.502	-0.804	-0.200	**0.002***
**Non-esterified cholesterol**				
Free cholesterol	-0.497	-0.761	-0.232	**<0.001***
Free cholesterol in chylomicrons and extremely large VLDL[b]	0.272	0.019	0.525	0.078
Free cholesterol in very large VLDL[b]	0.173	-0.072	0.418	0.361
Free cholesterol in large VLDL[b]	0.174	-0.067	0.416	0.337
Free cholesterol in medium VLDL	0.132	-0.146	0.410	0.746
Free cholesterol in small VLDL	-0.119	-0.369	0.129	0.728
Free cholesterol in very small VLDL	-0.419	-0.684	-0.154	**0.004***
Free cholesterol in IDL	-0.563	-0.841	-0.285	**<0.001***
Free cholesterol in large LDL	-0.549	-0.825	-0.273	**<0.001***
Free cholesterol in medium LDL	-0.515	-0.784	-0.247	**<0.001***
Free cholesterol in small LDL	-0.522	-0.788	-0.255	**<0.001***
Free cholesterol in very large HDL	-0.345	-0.615	-0.075	**0.023***
Free cholesterol in large HDL	-0.307	-0.574	-0.039	**0.048***
Free cholesterol in medium HDL	0.026	-0.267	0.320	1.637
Free cholesterol in small HDL	0.338	0.053	0.623	**0.038***
**Triglycerides**				
Serum total triglycerides	0.111	-0.166	0.388	2.258
Triglycerides in VLDL	0.201	-0.083	0.485	0.830
Triglycerides in chylomicrons and extremely large VLDL[b]	0.304	0.045	0.563	**0.039***
Triglycerides in very large VLDL[b]	0.243	-0.003	0.489	0.098
Triglycerides in large VLDL[b]	0.209	-0.035	0.455	0.169
Triglycerides in medium VLDL	0.223	-0.064	0.510	0.229
Triglycerides in small VLDL	0.083	-0.187	0.353	0.972
Triglycerides in very small VLDL	-0.098	-0.363	0.167	0.821
Triglycerides in IDL	-0.225	-0.498	0.048	0.520
Triglycerides in LDL	-0.273	-0.544	-0.002	0.229
Triglycerides in large LDL	-0.275	-0.549	-0.002	0.084
Triglycerides in medium LDL	-0.306	-0.577	-0.034	**0.047***
Triglycerides in small LDL	-0.202	-0.465	0.061	0.223
Triglycerides in HDL	-0.019	-0.287	0.249	4.110
Triglycerides in very large HDL	-0.168	-0.457	0.121	0.429
Triglycerides in large HDL	-0.257	-0.536	0.023	0.120
Triglycerides in medium HDL	0.178	-0.055	0.411	0.220
Triglycerides in small HDL	0.178	-0.088	0.445	0.309
**Phospholipids**				
Total phospholipids	-0.333	-0.579	-0.086	**0.036***
Phospholipids in VLDL	-0.018	-0.277	0.241	3.901
Phospholipids in chylomicrons and extremely large VLDL[b]	0.257	0.010	0.503	0.066
Phospholipids in very large VLDL[b]	0.189	-0.052	0.431	0.199
Phospholipids in large VLDL[b]	0.175	-0.066	0.417	0.246
Phospholipids in medium VLDL	0.129	-0.145	0.405	0.555
Phospholipids in small VLDL	-0.056	-0.302	0.191	1.022
Phospholipids in very small VLDL	-0.433	-0.698	-0.168	**0.002***
Phospholipids in IDL	-0.517	-0.788	-0.246	**<0.001***
Phospholipids in LDL	-0.466	-0.725	-0.206	**<0.001***
Phospholipids in large LDL	-0.493	-0.758	-0.229	**0.002***
Phospholipids in medium LDL	-0.428	-0.684	-0.173	**0.002***
Phospholipids in small LDL	-0.427	-0.679	-0.175	**<0.001***
Phospholipids in HDL	-0.109	-0.391	0.171	1.746
Phospholipids in very large HDL	-0.395	-0.663	-0.126	**0.006***

Phospholipids in large HDL	-0.277	-0.554	-0.001	0.072
Phospholipids in medium HDL	0.122	-0.165	0.410	0.587
Phospholipids in small HDL	0.452	0.167	0.738	**0.003***
Total phosphoglycerides	-0.308	-0.564	-0.052	0.077
Ratio of triglycerides to phosphoglycerides	0.287	-0.000	0.574	0.193
Phosphatidylcholine and other cholines	-0.375	-0.633	-0.117	**0.019***
Sphingomyelins	-0.491	-0.757	-0.224	**<0.001***
Total cholines	-0.406	-0.669	-0.142	**0.011***
**Fatty acids**				
Total fatty acids	-0.221	-0.472	0.030	0.122
Estimated degree of unsaturation	-0.256	-0.518	0.005	0.077
22:6, docosahexaenoic acid	-0.200	-0.479	0.078	0.223
18:2, linoleic acid	-0.458	-0.718	-0.199	**0.001***
Omega-3 fatty acids	-0.164	-0.433	0.106	0.321
Omega-6 fatty acids	-0.444	-0.703	-0.186	**0.001***
Polyunsaturated fatty acids	-0.417	-0.677	-0.156	**0.003***
Monounsaturated fatty acids; 16:1, 18:1	-0.089	-0.344	0.165	0.662
Saturated fatty acids	-0.128	-0.379	0.124	0.427
**Fatty acid ratios**				
22:6 docosahexaenoic acid to total fatty acids	-0.001	-0.245	0.243	1.305
18:2 linoleic acid to total fatty acids	-0.483	-0.751	-0.215	**<0.001***
Omega-3 fatty acids to total fatty acids	0.027	-0.208	0.262	1.071
Omega-6 fatty acids to total fatty acids	-0.478	-0.752	-0.204	**0.001***
Polyunsaturated fatty acids to total fatty acids	-0.407	-0.673	-0.142	**0.004***
Monounsaturated fatty acids to total fatty acids	0.323	0.055	0.591	**0.023***
Saturated fatty acids to total fatty acids	0.375	0.113	0.638	**0.006***
**Glycolysis related**				
Lactate	-0.054	-0.333	0.224	0.874
Pyruvate	0.282	0.026	0.538	**0.039***
Citrate	0.113	-0.153	0.378	0.498
**Amino acids**				
Alanine	0.047	-0.216	0.309	0.890
Glutamine	-0.254	-0.533	0.025	0.090
Glycine	-0.009	-0.297	0.279	1.147
Branched chain amino acids				
Isoleucine	0.448	0.172	0.724	**0.002***
Leucine	0.429	0.168	0.689	**0.001***
Valine	0.378	0.139	0.616	**0.002***
Aromatic amino acids				
Phenylalanine	0.226	-0.048	0.500	0.123
Tyrosine	0.316	0.059	0.573	**0.019***
Histidine	0.199	-0.061	0.459	0.155
**Ketone bodies**				
Acetoacetate[b]	0.350	0.067	0.633	**0.018***
3-hydroxybutyrate[b]	0.071	-0.200	0.342	0.691
**Inflammatory marker (NMR)**				
Glycoprotein acetlys, mainly a1-acid glycoprotein	0.142	-0.117	0.401	0.317
**Other (NMR)**				
Creatinine	-0.117	-0.352	0.118	0.368
Albumin	-0.134	-0.369	0.101	0.293
Acetate[b]	0.114	-0.123	0.351	0.381
**Conventionally measured analytes**				
**Glucose homeostasis**				
Insulin [b]	0.095	-0.124	0.314	0.429
C-peptide[b]	0.194	-0.017	0.406	0.078
Fructosamine	0.382	0.114	0.650	**0.006***
HOMA2-%B[b c]	-0.489	-0.712	-0.266	**<0.001***
HOMA2-%S[b c]	-0.153	-0.373	0.066	0.171
HOMA2-IR[b c]	0.150	-0.069	0.370	0.179
**Liver markers**				

gGT[b]	0.325	0.049	0.601	**0.023***
ALT[b]	0.094	-0.147	0.335	0.476
AST[b]	0.067	-0.182	0.316	0.638
SHBG	-0.247	-0.505	0.011	0.064
**Adipokines**				
Leptin[b]	-0.189	-0.430	0.053	0.131
Adiponectin[b]	-0.317	-0.577	-0.057	**0.018***
**Inflammatory markers**				
hs-CRP[b]	-0.157	-0.403	0.089	0.218
IL-6[b]	-0.215	-0.488	0.058	0.126
tPA-antigen[b]	0.046	-0.231	0.323	0.763
Ferritin[b]	0.067	-0.211	0.346	0.645

* False Discovery Rate (FDR) corrected *p* value <0.05 shown in bold [a] beta coefficient [b] analyte log transformed [c] insulin indices missing 7 in multivariate analysis (incalculable). GDM= gestational diabetes mellitus; gGT= γ-glutamyl transferase; ALT= alanine aminotransferase; AST= aspartate aminotransferase; SHBG= sex hormone binding globulin; hs-CRP= high sensitivity C-reactive protein; IL-6= interleukin-6; tPA-antigen= tissue plasminogen activator antigen; HOMA2-%B= steady state beta cell function; HOMA2-IR= insulin resistance; HOMA2-%S= insulin sensitivity; HDL= high-density lipoprotein; IDL=intermediate-density lipoproteins; LDL= low-density lipoprotein; VLDL= very low-density lipoproteins.
Data are adjusted for maternal BMI, parity, ethnicity, age, education and neonatal sex.

Table S5: Analyte concentrations (absolute units) at timepoint 1

Metabolites	No GDM<br>N= 112<br>Mean (SD)/Median (IQR)	GDM<br>N= 119<br>Mean (SD)/Median (IQR)
Total lipids		
Total lipids in chylomicrons and extremely large VLDL (umol/l)	22.0 (9.7-37.2)	22.5 (13.2-35.2)
Total lipids in very large VLDL (umol/l)	76.8 (39.7-110.3)	69.7 (46.1-116.9)
Total lipids in large VLDL (umol/l)	315.6 (191.8-427.4)	283.3 (206.1-429.3)
Total lipids in medium VLDL (mmol/l)	0.61 (0.23)	0.63 (0.25)
Total lipids in small VLDL (mmol/l)	0.64 (0.16)	0.64 (0.17)
Total lipids in very small VLDL (mmol/l)	0.55 (0.12)	0.52 (0.11)
Total lipids in IDL (mmol/l)	1.13 (0.26)	1.07 (0.23)
Total lipids in large LDL (mmol/l)	1.28 (0.31)	1.21 (0.28)
Total lipids in medium LDL (mmol/l)	0.72 (0.19)	0.68 (0.17)
Total lipids in small LDL (mmol/l)	0.47 (0.11)	0.44 (0.10)
Total lipids in very large HDL (mmol/l)	0.73 (0.19)	0.68 (0.19)
Total lipids in large HDL (mmol/l)	1.18 (0.28)	1.15 (0.29)
Total lipids in medium HDL (mmol/l)	0.99 (0.14)	1.02 (0.17)
Total lipids in small HDL (mmol/l)	1.12 (0.10)	1.16 (0.12)
**Particle diameter**		
Mean diameter for VLDL particles (nm)	36.9 (1.17)	37.0 (1.16)
Mean diameter for LDL particles (nm)	23.6 (0.07)	23.6 (0.06)
Mean diameter for HDL particles (nm)	10.2 (0.18)	10.2 (0.18)
**Apolipoproteins**		
Apolipoprotein A-1 (g/l)	1.77 (0.17)	1.76 (0.18)
Apolipoprotein B (g/l)	0.89 (0.16)	0.86 (0.15)
Ratio of apolipoprotein B to apolipoprotein A-1	0.50 (0.09)	0.49 (0.08)
**Cholesterol**		
Serum total cholesterol (mmol/l)	4.89 (0.85)	4.71 (0.81)
Total cholesterol in VLDL (mmol/l)	0.77 (0.19)	0.76 (0.19)
Total cholesterol in chylomicrons and extremely large VLDL (umol/l)	5.25 (3.1-7.5)	4.96 (3.2-7.4)
Total cholesterol in very large VLDL (umol/l)	15.5 (9.2-22.1)	14.3 (9.4-22.6)
Total cholesterol in large VLDL (umol/l)	70.7 (45.7-93.1)	64.3 (46.4-96.9)
Total cholesterol in medium VLDL (mmol/l)	0.17 (0.05)	0.17 (0.06)
Total cholesterol in small VLDL (mmol/l)	0.24 (0.05)	0.24 (0.06)
Total cholesterol in very small VLDL (mmol/l)	0.27 (0.06)	0.25 (0.05)
Remnant cholesterol (non-HDL, non-LDL -cholesterol) (mmol/l)	1.48 (0.33)	1.42 (0.30)
Total cholesterol in IDL (mmol/l)	0.69 (0.17)	0.66 (0.15)
Total cholesterol in LDL (mmol/l)	1.59 (0.46)	1.48 (0.41)
Total cholesterol in large LDL (mmol/l)	0.84 (0.23)	0.78 (0.20)
Total cholesterol in medium LDL (mmol/l)	0.46 (0.14)	0.43 (0.13)
Total cholesterol in small LDL (mmol/l)	0.28 (0.09)	0.26 (0.08)
Total cholesterol in HDL (mmol/l)	1.83 (0.27)	1.81 (0.29)
Total cholesterol in HDL2 (mmol/l)	1.28 (0.25)	1.26 (0.26)
Total cholesterol in HDL3 (mmol/l)	0.56 (0.03)	0.56 (0.04)
Total cholesterol in very large HDL (mmol/l)	0.34 (0.09)	0.31 (0.09)
Total cholesterol in large HDL (mmol/l)	0.57 (0.15)	0.55 (0.15)
Total cholesterol in medium HDL (mmol/l)	0.47 (0.08)	0.49 (0.09)
Total cholesterol in small HDL (mmol/l)	0.46 (0.06)	0.46 (0.06)
**Cholesteryl esters**		
Esterified cholesterol (mmol/l)	3.35 (0.65)	3.17 (0.59)
Cholesterol esters in chylomicrons and extremely large VLDL (umol/l)	3.09 (1.7-4.3)	2.98 (1.8-4.2)
Cholesterol esters in very large VLDL (umol/l)	8.61 (5.4-12.4)	8.13 (5.5-12.5)
Cholesteryl esters in large VLDL (umol/l)	35.6 (25.7-47.2)	34.5 (25.6-49.7)

Cholesteryl esters in medium VLDL (mmol/l)	0.09 (0.03)	0.09 (0.03)
Cholesteryl esters in small VLDL (mmol/l)	0.15 (0.04)	0.14 (0.03)
Cholesteryl esters in very small VLDL (mmol/l)	0.18 (0.04)	0.17 (0.03)
Cholesteryl esters in IDL (mmol/l)	0.50 (0.12)	0.47 (0.11)
Cholesteryl esters in large LDL (mmol/l)	0.59 (0.17)	0.56 (0.15)
Cholesteryl esters in medium LDL (mmol/l)	0.32 (0.12)	0.29 (0.11)
Cholesteryl esters in small LDL (mmol/l)	0.19 (0.07)	0.18 (0.06)
Cholesteryl esters in very large HDL (mmol/l)	0.24 (0.06)	0.23 (0.06)
Cholesteryl esters in large HDL (mmol/l)	0.44 (0.11)	0.43 (0.11)
Cholesteryl esters in medium HDL (mmol/l)	0.38 (0.06)	0.39 (0.07)
Cholesteryl esters in small HDL (mmol/l)	0.34 (0.05)	0.34 (0.05)
**Non-esterified cholesterol**		
Free cholesterol (mmol/l)	1.53 (0.28)	1.46 (0.26)
Free cholesterol in chylomicrons and extremely large VLDL (umol/l)	2.24 (1.0-3.4)	2.17 (1.4-3.2)
Free cholesterol in very large VLDL (umol/l)	6.92 (3.7- 10.2)	6.39 (4.3-10.1)
Free cholesterol in large VLDL (umol/l)	34.5 (19.6-46.8)	31.1 (20.8-47.8)
Free cholesterol in medium VLDL (mmol/l)	0.07 (0.03)	0.08 (0.03)
Free cholesterol in small VLDL (mmol/l)	0.09 (0.02)	0.09 (0.02)
Free cholesterol in very small VLDL (mmol/l)	0.09 (0.02)	0.08 (0.02)
Free cholesterol in IDL (mmol/l)	0.19 (0.05)	0.18 (0.05)
Free cholesterol in large LDL (mmol/l)	0.24 (0.06)	0.23 (0.05)
Free cholesterol in medium LDL (mmol/l)	0.14 (0.02)	0.13 (0.02)
Free cholesterol in small LDL (mmol/l)	0.08 (0.01)	0.08 (0.01)
Free cholesterol in very large HDL (mmol/l)	0.09 (0.03)	0.09 (0.02)
Free cholesterol in large HDL (mmol/l)	0.12 (0.03)	0.12 (0.03)
Free cholesterol in medium HDL (mmol/l)	0.09 (0.02)	0.09 (0.02)
Free cholesterol in small HDL (mmol/l)	0.12 (0.01)	0.12 (0.01)
**Triglycerides**		
Serum total triglycerides (mmol/l)	1.48 (0.44)	1.49 (0.48)
Triglycerides in VLDL (mmol/l)	0.94 (0.37)	0.96 (0.41)
Triglycerides in chylomicrons and extremely large VLDL (umol/l)	13.4 (5.9-25.2)	14.6 (7.8-23.4)
Triglycerides in very large VLDL (umol/l)	47.1 (25.0-70.7)	46.7 (28.3-71.9)
Triglycerides in large VLDL (umol/l)	183.3 (107.0-253.9)	173.4 (119.5-258.6)
Triglycerides in medium VLDL (mmol/l)	0.32 (0.13)	0.33 (0.15)
Triglycerides in small VLDL (mmol/l)	0.25 (0.08)	0.25 (0.08)
Triglycerides in very small VLDL (mmol/l)	0.12 (0.03)	0.12 (0.03)
Triglycerides in IDL (mmol/l)	0.14 (0.03)	0.13 (0.03)
Triglycerides in LDL (mmol/l)	0.22 (0.06)	0.21 (0.05)
Triglycerides in large LDL (mmol/l)	0.12 (0.03)	0.12 (0.03)
Triglycerides in medium LDL (mmol/l)	0.06 (0.01)	0.06 (0.01)
Triglycerides in small LDL (mmol/l)	0.04 (0.01)	0.04 (0.01)
Triglycerides in HDL (mmol/l)	0.18 (0.03)	0.18 (0.04)
Triglycerides in very large HDL (mmol/l)	0.03 (0.01)	0.03 (0.01)
Triglycerides in large HDL (mmol/l)	0.05 (0.01)	0.05 (0.02)
Triglycerides in medium HDL (mmol/l)	0.05 (0.01)	0.05 (0.01)
Triglycerides in small HDL (mmol/l)	0.05 (0.01)	0.05 (0.01)
**Phospholipids**		
Total phospholipids (mmol/l)	3.47 (0.38)	3.43 (0.40)
Phospholipids in VLDL (mmol/l)	0.50 (0.13)	0.50 (0.14)
Phospholipids in chylomicrons and extremely large VLDL (umol/l)	3.06 (1.3-4.9)	3.05 (1.6-4.7)
Phospholipids in very large VLDL (umol/l)	12.6 (6.5-18.2)	11.6 (7.5-19.1)
Phospholipids in large VLDL (umol/l)	58.3 (37.1-77.7)	53.9 (38.1-79.4)
Phospholipids in medium VLDL (mmol/l)	0.12 (0.04)	0.12 (0.05)
Phospholipids in small VLDL (mmol/l)	0.15 (0.03)	0.15 (0.04)
Phospholipids in very small VLDL (mmol/l)	0.15 (0.04)	0.15 (0.03)
Phospholipids in IDL (mmol/l)	0.29 (0.06)	0.28 (0.06)
Phospholipids in LDL (mmol/l)	0.66 (0.12)	0.64 (0.11)
Phospholipids in large LDL (mmol/l)	0.15 (0.02)	0.14 (0.02)
Phospholipids in medium LDL (mmol/l)	0.19 (0.03)	0.19 (0.03)

Phospholipids in small LDL (mmol/l)	0.32 (0.06)	0.30 (0.6)
Phospholipids in HDL (mmol/l)	2.00 (0.27)	2.01 (0.29)
Phospholipids in very large HDL (mmol/l)	0.36 (0.10)	0.34 (0.10)
Phospholipids in large HDL (mmol/l)	0.56 (0.12)	0.55 (0.13)
Phospholipids in medium HDL (mmol/l)	0.47 (0.06)	0.48 (0.08)
Phospholipids in small HDL (mmol/l)	0.61 (0.07)	0.64 (0.09)
Total phosphoglycerides (mmol/l)	2.66 (0.33)	2.60 (0.31)
Ratio of triglycerides to phosphoglycerides (mmol/l)	0.65 (0.16)	0.66 (0.17)
Phosphatidylcholine and other cholines (mmol/l)	2.42 (0.32)	2.34 (0.31)
Sphingomyelins (mmol/l)	0.46 (0.08)	0.44 (0.08)
Total cholines (mmol/l)	3.02 (0.35)	2.93 (0.34)
**Fatty acids**		
Total fatty acids (mmol/l)	14.7 (2.08)	14.5 (2.17)
Estimated degree of unsaturation	1.12 (0.04)	1.12 (0.05)
22:6, docosahexaenoic acid (mmol/l)	0.24 (0.04)	0.24 (0.05)
18:2, linoleic acid (mmol/l)	3.71 (0.62)	3.56 (0.59)
Omega-3 fatty acids (mmol/l)	0.70 (0.13)	0.69 (0.16)
Omega-6 fatty acids (mmol/l)	4.34 (0.64)	4.22 (0.63)
Polyunsaturated fatty acids (mmol/l)	5.05 (0.73)	4.92 (0.75)
Monounsaturated fatty acids; 16:1, 18:1 (mmol/l)	4.18 (0.76)	4.12 (0.79)
Saturated fatty acids (mmol/l)	5.51 (0.80)	5.43 (0.84)
**Fatty acid ratios**		
22:6 docosahexaenoic acid to total fatty acids (%)	1.64 (0.26)	1.68 (0.30)
18:2 linoleic acid to total fatty acids (%)	25.2 (2.61)	24.7 (2.60)
Omega-3 fatty acids to total fatty acids (%)	4.80 (0.71)	4.84 (0.82)
Omega-6 fatty acids to total fatty acids (%)	29.6 (2.59)	29.3 (2.51)
Polyunsaturated fatty acids to total fatty acids (%)	34.4 (2.96)	34.1 (2.92)
Monounsaturated fatty acids to total fatty acids (%)	28.2 (2.17)	28.3 (2.13)
Saturated fatty acids to total fatty acids (%)	37.4 (1.32)	37.5 (1.30)
**Glycolysis related**		
Glucose (mmol/l)	4.04 (0.66)	4.34 (0.86)
Lactate (mmol/l)	1.31 (0.39)	1.38 (0.49)
Pyruvate (umol/l)	102.6 (30.01)	113.7 (52.75)
Citrate (umol/l)	113.1 (18.98)	113.1 (17.95)
**Amino acids**		
Alanine (umol/l)	391.4 (35.23)	386.7 (39.65)
Glutamine (umol/l)	373.8 (42.59)	357.0 (45.73)
Glycine (umol/l)	218.4 (21.97)	214.5 (23.21)
Branched chain amino acids		
Isoleucine (umol/l)	50.8 (13.97)	51.9 (12.79)
Leucine (umol/l)	67.1 (14.59)	68.9 (13.23)
Valine (umol/l)	137.2 (32.97)	141.4 (26.04)
Aromatic amino acids		
Phenylalanine (umol/l)	76.0 (10.35)	78.6 (10.56)
Tyrosine (umol/l)	42.8 (9.51)	42.8 (8.11)
Histidine (umol/l)	67.4 (6.70)	66.1 (5.62)
**Ketone bodies**		
Acetoacetate (umol/l)	23.3 (16.6-29.4)	22.9 (18.2-33.4)
3-hydroxybutyrate (umol/l)	86.7 (70.1-114.3)	94.5 (73.2-120.4)
**Inflammatory marker (NMR)**		
Glycoprotein acetlys, mainly a1-acid glycoprotein (mmol/l)	1.52 (0.12)	1.55 (0.16)
**Other (NMR)**		
Creatinine (mmol/l)	0.04 (0.00)	0.04 (0.00)
Albumin (signal area)	0.08 (0.00)	0.08 (0.00)
Acetate	39.2 (35.3-43.9)	39.7 (36.2-43.6)
**Conventionally measured analytes**		
**Glucose homeostasis**		
HbA1c (%)	5.04 (0.31)	5.16 (0.35)
HbA1c (mmol/mol)	31.5 (3.45)	32.9 (3.87)
Insulin (Uu/ml)	40.6 (15.9-71.7)	52.9 (24.7-103.1)

C-peptide (ng/ml)	4.98 (3.1- 7.7)	6.61 (3.9-8.7)
Fructosamine (umol/l)	189.3 (21.65)	194.4 (23.48)
**Liver markers**		
gGT (U/L)	16.2 (12.19)	21.7 (17.73)
ALT (U/L)	15.7 (11.9-22.6)	16.9 (12.5-24.2)
AST (U/L)	21.8 (17.8-27.9)	23.7 (19.2-29.2)
SHBG (nmol/l)	417.2 (128.09)	372.4 (111.78)
**Adipokines**		
Leptin (pg/ml)	74.9 (48.6-94.8)	67.8 (47.4-91.9)
Adiponectin (ng/ml)	8.99 (6.8-13.0)	6.70 (3.8-11.5)
**Inflammatory markers**		
hs-CRP (mg/l)	7.31 (5.3-11.8)	7.52 (5.2-12.2)
IL-6 (pg/ml)	3.46 (2.4-5.2)	3.01 (1.9-4.9)
tPA-antigen (ng/ml)	6.95 (5.6-9.4)	7.85 (5.7-10.9)
Ferritin (pmol/l)	42.1 (24.8-77.6)	53.2 (30.9-82.9)
**Miscellaneous**		
Vitamin D (nmol/l)	15.1 (10.0-22.6)	15.0 (8.7-19.5)
hPL (z score)	-0.09 (1.06)	-0.38 (0.90)

GDM= gestational diabetes mellitus; gGT= γ-glutamyl transferase; ALT= alanine aminotransferase; AST= aspartate aminotransferase, SHBG= sex hormone binding globulin; hs-CRP= high sensitivity C-reactive protein; IL-6= interleukin-6; tPA-antigen= tissue plasminogen activator antigen; hPL= human placental lactogen

Table S6: Analyte concentrations (absolute units) at timepoint 2

Metabolites	No GDM<br>N= 112<br>Mean (SD)/Median (IQR)	GDM<br>N= 119<br>Mean (SD)/Median (IQR)
**Total lipids**		
Total lipids in chylomicrons and extremely large VLDL (umol/l)	24.5 (13.7-38.5)	24.3 (12.5-48.1)
Total lipids in very large VLD (umol/l)	90.5 (56.1-134.4)	93.5 (51.8-159.5)
Total lipids in large VLDL (umol/l)	384.6 (267.8-536.7)	387.7 (235.3-614.2)
Total lipids in medium VLDL (mmol/l)	0.78 (0.31)	0.79 (0.35)
Total lipids in small VLDL (mmol/l)	0.80 (0.22)	0.77 (0.21)
Total lipids in very small VLDL (mmol/l)	0.67 (0.16)	0.62 (0.12)
Total lipids in IDL (mmol/l)	1.36 (0.34)	1.22 (0.26)
Total lipids in large LDL (mmol/l)	1.55 (0.41)	1.38 (0.32)
Total lipids in medium LDL (mmol/l)	0.89 (0.25)	0.78 (0.19)
Total lipids in small LDL (mmol/l)	0.57 (0.15)	0.51 (0.11)
Total lipids in very large HDL (mmol/l)	0.81 (0.20)	0.74 (0.21)
Total lipids in large HDL (mmol/l)	1.23 (0.28)	1.17 (0.29)
Total lipids in medium HDL (mmol/l)	0.95 (0.16)	0.97 (0.16)
Total lipids in small HDL (mmol/l)	1.12 (0.10)	1.13 (0.11)
**Particle diameter**		
Mean diameter for VLDL particles (nm)	36.9 (1.06)	37.2 (1.23)
Mean diameter for LDL particles (nm)	23.6 (0.05)	23.6 (0.07)
Mean diameter for HDL particles (nm)	10.3 (0.19)	10.2 (0.19)
**Apolipoproteins**		
Apolipoprotein A-1 (g/l)	1.84 (0.17)	1.79 (0.17)
Apolipoprotein B (g/l)	1.06 (0.22)	0.99 (0.18)
Ratio of apolipoprotein B to apolipoprotein A-1	0.58 (0.12)	0.56 (0.10)
**Cholesterol**		
Serum total cholesterol (mmol/l)	5.63 (1.06)	5.17 (0.85)
Total cholesterol in VLDL (mmol/l)	0.97 (0.26)	0.92 (0.23)
Total cholesterol in chylomicrons and extremely large VLDL (umol/l)	6.68 (4.7-9.3)	6.71 (3.8-10.4)
Total cholesterol in very large VLDL (umol/l)	19.1 (12.7-27.1)	20.1 (10.8-31.3)
Total cholesterol in large VLDL (umol/l)	87.9 (61.2-120.1)	90.1 (55.9-135.6)
Total cholesterol in medium VLDL (mmol/l)	0.22 (0.08)	0.22 (0.08)
Total cholesterol in small VLDL (mmol/l)	0.30 (0.08)	0.28 (0.06)
Total cholesterol in very small VLDL (mmol/l)	0.32 (0.08)	0.29 (0.06)
Remnant cholesterol (non-HDL, non-LDL -cholesterol) (mmol/l)	1.80 (0.44)	1.67 (0.35)
Total cholesterol in IDL (mmol/l)	0.84 (0.22)	0.75 (0.17)
Total cholesterol in LDL (mmol/l)	1.95 (0.60)	1.69 (0.47)
Total cholesterol in large LDL (mmol/l)	1.02 (0.29)	0.89 (0.23)
Total cholesterol in medium LDL (mmol/l)	0.58 (0.19)	0.49 (0.15)
Total cholesterol in small LDL (mmol/l)	0.35 (0.11)	0.30 (0.09)
Total cholesterol in HDL (mmol/l)	1.87 (0.29)	1.80 (0.29)
Total cholesterol in HDL2 (mmol/l)	1.29 (0.27)	1.23 (0.26)
Total cholesterol in HDL3 (mmol/l)	0.58 (0.03)	0.57 (0.03)
Total cholesterol in very large HDL (mmol/l)	0.37 (0.09)	0.34 (0.09)
Total cholesterol in large HDL (mmol/l)	0.59 (0.15)	0.56 (0.15)
Total cholesterol in medium HDL (mmol/l)	0.44 (0.09)	0.44 (0.09)
Total cholesterol in small HDL (mmol/l)	0.48 (0.06)	0.46 (0.05)
**Cholesteryl esters**		
Esterified cholesterol (mmol/l)	3.55 (0.75)	3.22 (0.61)
Cholesterol esters in chylomicrons and extremely large VLDL (umol/l)	3.92 (2.8-5.3)	3.85 (2.4-5.5)
Cholesterol esters in very large VLDL (umol/l)	10.7 (7.2-14.8)	10.8 (6.3-16.8)
Cholesteryl esters in large VLDL (umol/l)	47.3 (32.1-60.6)	45.7 (29.5-65.9)
Cholesteryl esters in medium VLDL (mmol/l)	0.13 (0.04)	0.12 (0.04)

Cholesteryl esters in small VLDL (mmol/l)	0.18 (0.05)	0.16 (0.04)
Cholesteryl esters in very small VLDL (mmol/l)	0.21 (0.05)	0.19 (0.04)
Cholesteryl esters in IDL (mmol/l)	0.59 (0.15)	0.54 (0.12)
Cholesteryl esters in large LDL (mmol/l)	0.74 (0.22)	0.64 (0.17)
Cholesteryl esters in medium LDL (mmol/l)	0.42 (0.16)	0.35 (0.12)
Cholesteryl esters in small LDL (mmol/l)	0.25 (0.09)	0.21 (0.07)
Cholesteryl esters in very large HDL (mmol/l)	0.26 (0.07)	0.25 (0.07)
Cholesteryl esters in large HDL (mmol/l)	0.46 (0.11)	0.44 (0.12)
Cholesteryl esters in medium HDL (mmol/l)	0.34 (0.07)	0.35 (0.07)
Cholesteryl esters in small HDL (mmol/l)	0.36 (0.06)	0.34 (0.05)
**Non-esterified cholesterol**		
Free cholesterol (mmol/l)	1.55 (0.32)	1.42 (0.24)
Free cholesterol in chylomicrons and extremely large VLDL (umol/l)	2.64 (1.5-3.9)	2.73 (1.4-4.7)
Free cholesterol in very large VLDL (umol/l)	8.45 (5.7-12.2)	9.09 (4.7-14.3)
Free cholesterol in large VLDL (umol/l)	42.2 (29.7-62.7)	42.9 (25.6-69.1)
Free cholesterol in medium VLDL (mmol/l)	0.09 (0.04)	0.10 (0.04)
Free cholesterol in small VLDL (mmol/l)	0.12 (0.03)	0.12 (0.03)
Free cholesterol in very small VLDL (mmol/l)	0.11 (0.03)	0.09 (0.02)
Free cholesterol in IDL (mmol/l)	0.24 (0.07)	0.021 (0.05)
Free cholesterol in large LDL (mmol/l)	0.28 (0.07)	0.25 (0.06)
Free cholesterol in medium LDL (mmol/l)	0.16 (0.03)	0.15 (0.03)
Free cholesterol in small LDL (mmol/l)	0.09 (0.02)	0.09 (0.01)
Free cholesterol in very large HDL (mmol/l)	0.10 (0.03)	0.09 (0.03)
Free cholesterol in large HDL (mmol/l)	0.13 (0.03)	0.12 (0.04)
Free cholesterol in medium HDL (mmol/l)	0.09 (0.02)	0.09 (0.02)
Free cholesterol in small HDL (mmol/l)	0.11 (0.01)	0.12 (0.01)
**Triglycerides**		
Serum total triglycerides (mmol/l)	1.88 (0.59)	1.88 (0.67)
Triglycerides in VLDL (mmol/l)	1.19 (0.48)	1.23 (0.57)
Triglycerides in chylomicrons and extremely large VLDL (umol/l)	14.3 (7.6-24.6)	14.8 (6.2-30.5)
Triglycerides in very large VLDL (umol/l)	57.7 (33.9-84.8)	59.7 (30.9-101.6)
Triglycerides in large VLDL (umol/l)	227.1 (153.3-315.2)	223.9 (137.2-361.8)
Triglycerides in medium VLDL (mmol/l)	0.39 (0.17)	0.42 (0.20)
Triglycerides in small VLDL (mmol/l)	0.31 (0.11)	0.31 (0.11)
Triglycerides in very small VLDL (mmol/l)	0.16 (0.05)	0.15 (0.04)
Triglycerides in IDL (mmol/l)	0.18 (0.05)	0.17 (0.04)
Triglycerides in LDL (mmol/l)	0.29 (0.08)	0.27 (0.06)
Triglycerides in large LDL (mmol/l)	0.16 (0.04)	0.15 (0.3)
Triglycerides in medium LDL (mmol/l)	0.08 (0.02)	0.07 (0.02)
Triglycerides in small LDL (mmol/l)	0.05 (0.01)	0.04 (0.01)
Triglycerides in HDL (mmol/l)	0.21 (0.04)	0.21 (0.04)
Triglycerides in very large HDL (mmol/l)	0.03 (0.01)	0.03 (0.01)
Triglycerides in large HDL (mmol/l)	0.06 (0.01)	0.06 (0.02)
Triglycerides in medium HDL (mmol/l)	0.05 (0.01)	0.05 (0.01)
Triglycerides in small HDL (mmol/l)	0.06 (0.01)	0.06 (0.01)
**Phospholipids**		
Total phospholipids (mmol/l)	3.78 (0.39)	3.63 (0.38)
Phospholipids in VLDL (mmol/l)	0.64 (0.18)	0.62 (0.18)
Phospholipids in chylomicrons and extremely large VLDL (umol/l)	3.52 (1.9-5.5)	3.59 (1.9-6.7)
Phospholipids in very large VLDL (umol/l)	15.3 (9.6-22.5)	15.4 (8.3-26.3)
Phospholipids in large VLDL (umol/l)	71.1 (50.8-101.6)	72.8 (44.6-112.6)
Phospholipids in medium VLDL (mmol/l)	0.16 (0.06)	0.16 (0.07)
Phospholipids in small VLDL (mmol/l)	0.18 (0.05)	0.18 (0.04)
Phospholipids in very small VLDL (mmol/l)	0.19 (0.05)	0.17 (0.04)
Phospholipids in IDL (mmol/l)	0.34 (0.08)	0.31 (0.07)
Phospholipids in LDL (mmol/l)	0.77 (0.15)	0.71 (0.12)
Phospholipids in large LDL (mmol/l)	0.37 (0.08)	0.34 (0.06)
Phospholipids in medium LDL (mmol/l)	0.23 (0.04)	0.21 (0.03)
Phospholipids in small LDL (mmol/l)	0.17 (0.03)	0.16 (0.02)

Phospholipids in HDL (mmol/l)	2.03 (0.27)	1.99 (0.28)
Phospholipids in very large HDL (mmol/l)	0.40 (0.11)	0.37 (0.11)
Phospholipids in large HDL (mmol/l)	0.58 (0.12)	0.55 (0.12)
Phospholipids in medium HDL (mmol/l)	0.46 (0.07)	0.47 (0.07)
Phospholipids in small HDL (mmol/l)	0.58 (0.08)	0.61 (0.09)
Total phosphoglycerides (mmol/l)	2.67 (0.32)	2.56 (0.33)
Ratio of triglycerides to phosphoglycerides (mmol/l)	0.72 (0.18)	0.75 (0.19)
Phosphatidylcholine and other cholines (mmol/l)	2.45 (0.33)	2.32 (0.31)
Sphingomyelins (mmol/l)	0.43 (0.08)	0.39 (0.07)
Total cholines (mmol/l)	2.89 (0.36)	2.76 (0.34)
**Fatty acids**		
Total fatty acids (mmol/l)	14.9 (2.27)	14.3 (2.18)
Estimated degree of unsaturation	1.09 (0.04)	1.09 (0.05)
22:6, docosahexaenoic acid (mmol/l)	0.22 (0.04)	0.22 (0.04)
18:2, linoleic acid (mmol/l)	3.72 (0.69)	3.43 (0.56)
Omega-3 fatty acids (mmol/l)	0.67 (0.13)	0.66 (0.13)
Omega-6 fatty acids (mmol/l)	4.28 (0.73)	3.99 (0.60)
Polyunsaturated fatty acids (mmol/l)	4.96 (0.83)	4.64 (0.70)
Monounsaturated fatty acids; 16:1, 18:1 (mmol/l)	4.52 (0.75)	4.39 (0.80)
Saturated fatty acids (mmol/l)	5.47 (0.84)	5.29 (0.87)
**Fatty acid ratios**		
22:6 docosahexaenoic acid to total fatty acids (%)	1.51 (0.23)	1.54 (0.28)
18:2 linoleic acid to total fatty acids (%)	24.8 (2.14)	24.0 (2.43)
Omega-3 fatty acids to total fatty acids (%)	4.51 (0.64)	4.61 (0.77)
Omega-6 fatty acids to total fatty acids (%)	28.7 (2.10)	27.9 (2.48)
Polyunsaturated fatty acids to total fatty acids (%)	33.2 (2.45)	32.5 (2.96)
Monounsaturated fatty acids to total fatty acids (%)	30.2 (1.62)	30.6 (1.84)
Saturated fatty acids to total fatty acids (%)	36.6 (1.25)	36.9 (1.43)
**Glycolysis related**		
Glucose (mmol/l)	3.81 (0.72)	4.31 (0.89)
Lactate (mmol/l)	1.46 (0.55)	1.42 (0.42)
Pyruvate (umol/l)	98.2 (38.77)	108.2 (30.61)
Citrate (umol/l)	119.6 (15.13)	121.4 (16.85)
**Amino acids**		
Alanine (umol/l)	389.0 (37.11)	387.5 (38.47)
Glutamine (umol/l)	368.3 (38.61)	357.4 (40.17)
Glycine (umol/l)	222.3 (27.38)	221.9 (24.33)
Branched chain amino acids		
Isoleucine (umol/l)	47.7 (9.69)	52.1 (11.28)
Leucine (umol/l)	61.3 (9.16)	65.9 (11.01)
Valine (umol/l)	118.6 (19.02)	128.1 (18.37)
Aromatic amino acids		
Phenylalanine (umol/l)	79.1 (10.59)	81.3 (10.44)
Tyrosine (umol/l)	35.9 (5.74)	37.9 (5.62)
Histidine (umol/l)	64.7 (5.86)	65.8 (5.70)
**Ketone bodies**		
Acetoacetate (umol/l)	25.1 (19.7-30.9)	28.1 (22.0-35.9)
3-hydroxybutyrate (umol/l)	126.8 (98.8-155.4)	122.8 (108.1-153.0)
**Inflammatory marker (NMR)**		
Glycoprotein acetlys, mainly a1-acid glycoprotein (mmol/l)	1.62 (0.15)	1.63 (0.16)
**Other (NMR)**		
Creatinine (mmol/l)	0.04 (0.01)	0.04 (0.00)
Albumin (signal area)	0.08 (0.00)	0.08 (0.00)
Acetate (umol/l)	41.8 (37.2-46.5)	41.7 (37.8-48.5)
**Conventionally measured analytes**		
**Glucose homeostasis**		
Insulin (Uu/ml)	17.6 (13.0-28.2)	21.9 (15.7-30.9)
C-peptide (ng/ml)	3.27 (2.7-4.7)	3.79 (3.0-4.8)
Fructosamine (umol/l)	178.3 (13.89)	185.0 (16.43)
**Insulin indices** [a]		

HOMA2-%B	220.8 (175.1-294.6)	189.3 (156.7-227.0)
HOMA2-%S	3.69 (3.2-3.9)	3.44 (3.1-3.7)
HOMA2-IR	2.50 (1.8-3.9)	3.20 (2.3-4.3)
**Liver markers**		
gGT (U/L)	15.1 (10.86)	20.9 (18.53)
ALT (U/L)	13.9 (10.0-17.4)	13.8 (10.4-18.3)
AST (U/L)	20.8 (16.9-24.6)	22.1 (18.0-26.5)
SHBG (nmol/l)	503.4 (144.56)	470.6 (136.64)
**Adipokines**		
Leptin (pg/ml)	71.1 (51.3-91.4)	60.6 (43.3-87.3)
Adiponectin (ng/ml)	8.85 (6.6-12.6)	6.84 (4.2-10.4)
**Inflammatory markers**		
hs-CRP (mg/l)	7.10 (4.9-11.4)	6.84 (4.6-10.8)
IL-6 (pg/ml)	2.18 (1.6-2.9)	2.05 (1.6-2.5)
tPA-antigen (ng/ml)	7.41 (5.7-10.5)	8.60 (5.9-12.8)
Ferritin (pmol/l)	21.8 (11.5-33.7)	20.5 (13.6-37.1)

GDM= gestational diabetes mellitus; gGT= γ-glutamyl transferase; ALT= alanine aminotransferase; AST= aspartate aminotransferase, SHBG= sex hormone binding globulin; hs-CRP= high sensitivity C-reactive protein; IL-6= interleukin-6; tPA-antigen= tissue plasminogen activator antigen; HOMA2-%B= steady state beta cell function; HOMA2-IR= insulin resistance; HOMA2-%S= insulin sensitivity. [a] Missing insulin index as incalculable: 7
